# Telomerase Mediates Lymphocyte Proliferation but Not the Atherosclerosis-Suppressive Potential of Regulatory T-Cells

**DOI:** 10.1161/ATVBAHA.117.309940

**Published:** 2018-05-29

**Authors:** Gavin David Richardson, Andrew Sage, Karim Bennaceur, Nayef Al Zhrany, Jose Coelho-Lima, Emily Dookun, Lilia Draganova, Gabriele Saretzki, David T. Breault, Ziad Mallat, Ioakim Spyridopoulos

**Affiliations:** 1 From the Cardiovascular Research Centre, Institute of Genetic Medicine, International Centre for Life (G.D.R., K.B., N.A.Z., J.C.-L., E.D., L.D., I.S.); 2 Institute for Cell and Molecular Biosciences, The Ageing Biology Centre, Newcastle University Institute for Ageing, Campus for Ageing and Vitality (G.S.), Newcastle University, Newcastle upon Tyne, United Kingdom; 3 Division of Cardiovascular Medicine, Department of Medicine, University of Cambridge, United Kingdom (A.S., Z.M.); 4 Division of Endocrinology, Boston Children’s Hospital, Harvard Medical School, MA (D.T.B.); 5 Harvard Stem Cell Institute, Cambridge, MA (D.T.B.); 6 INSERM U970, Paris Cardiovascular Research Center, France (Z.M.); 7 Université Paris Descartes, Sorbonne Paris Cité, France (Z.M.).

**Keywords:** atherosclerosis, lymphocytes, models, animal, oxidative stress, telomerase

## Abstract

**Objective—:**

Atherosclerosis is an age-related disease characterized by systemic oxidative stress and low-grade inflammation. The role of telomerase and telomere length in atherogenesis remains contentious. Short telomeres of peripheral leukocytes are predictive for coronary artery disease. Conversely, attenuated telomerase has been demonstrated to be protective for atherosclerosis. Hence, a potential causative role of telomerase in atherogenesis is critically debated.

**Approach and Results—:**

In this study, we used multiple mouse models to investigate the regulation of telomerase under oxidative stress as well as its impact on atherogenesis in vitro and in vivo. Using primary lymphocytes and myeloid cell cultures, we demonstrate that cultivation under hyperoxic conditions induced oxidative stress resulting in chronic activation of CD4^+^ cells and significantly reduced CD4^+^ T-cell proliferation. The latter was telomerase dependent because oxidative stress had no effect on the proliferation of primary lymphocytes isolated from telomerase knockout mice. In contrast, myeloid cell proliferation was unaffected by oxidative stress nor reliant on telomerase. Telomerase reverse transcriptase deficiency had no effect on regulatory T-cell (T_reg_) numbers in vivo or suppressive function ex vivo. Adoptive transfer of telomerase reverse transcriptase^–^^/–^ T_regs_ into Rag2^–/–^ ApoE^–/–^ (recombination activating gene 2/apolipoprotein E) double knockout mice demonstrated that telomerase function was not required for the ability of T_regs_ to protect against atherosclerosis. However, telomere length was critical for T_reg_ function.

**Conclusions—:**

Telomerase contributes to lymphocyte proliferation but plays no major role in T_reg_ function, provided that telomere length is not critically short. We suggest that oxidative stress may contribute to atherosclerosis via suppression of telomerase and acceleration of telomere attrition in T_regs_.

## Abstract 2

Supplemental Digital Content is available in the text.

Atherosclerosis is an age-related systemic disease characterized by systemic oxidative stress and low-grade chronic inflammation, which is driven both by innate and adaptive immune responses. In particular, distinct subpopulations of T-cells have opposing roles in the development of atherosclerotic lesions,^1^ and an imbalance between pathogenic and regulatory immunity influences plaque development and disease progression. We and others have shown previously that the majority of T-cells in an atherosclerotic lesion are CD4^+^ T-helper type 1 cells that produce interferon-γ^2–12^ and deficiency in either interferon-γ or the transcription factor T-bet (required for T-helper type 1 differentiation) attenuates the progression of atherosclerosis in cholesterol-fed low-density lipoprotein receptor null mice (*Ldlr*^−/−^). Conversely, reduction or dysfunction of the regulatory T-cell population (T_reg_) leads to increased atherosclerosis.^5,13,14^ Furthermore, adoptive transfer of the T_reg_ subpopulation into hypercholesterolemic mice reduces lesion development.^5^ Recently, it has also been demonstrated that prolonged hypercholesterolemia impairs T_reg_ cells but not effector T-cell accumulation in atherosclerotic lesions and reversal of hypercholesterolemia can prevent loss of lesional T_reg_ cells.^12^ The ratio of regulatory to effector T-cells is, therefore, critical in determining the outcome of atherosclerosis.

**See accompanying editorial on page 1247**

Human T_reg_ cells do not arise solely from thymic generation but can also be induced by rapid turnover from the memory T-cell pool.^15^ Once generated, they are susceptible to apoptosis and have limited replicative potential, which is directly related to telomere length.^16^ Telomeres consist of tandem TTAGGG DNA repeats at the ends of chromosomes as well as the shelterin complex. They function to maintain chromosomal integrity during cell division and protect against chromosomal instability. In cells without telomerase activity, telomeres shorten with every cell division because of the end replication problem,^17^ as well as because of increased oxidative stress.^18^ Telomere shortening can be compensated or slowed down by concomitant activity of telomerase, a ribonucleoprotein composed of an RNA subunit (telomerase RNA component [TERC]), containing the template for telomere repeat addition, and a telomerase reverse transcriptase (TERT) subunit. Accordingly, late-generation telomerase knockout mice (TERT^−/−^ or TERC^−/−^) display an age-related phenotype.^19–21^

Regarding its specific role in T-lymphocytes, telomerase levels control the lifespan of T lymphocytes^22^ while telomere dysfunction in late-generation TERC^−/−^ mice reduces the number and function of T- and B- lymphocytes.^16,20,23,24^ The end replication problem is not the only mechanism that can contribute to telomere shortening resulting eventually in cellular senescence. Telomeres have been shown to be favored targets for DNA damage.^25,26^ Because oxidative stress plays a major role in chronic inflammatory diseases, it has been suggested that telomere damage may be involved in their pathophysiology. However, the exact mechanisms driving telomere damage and shortening under conditions of chronic oxidative stress are still not fully understood.

Telomere length in leukocytes has been investigated in a variety of clinical studies involving patients with either existing or developing coronary heart disease (CHD).^27–33^ There remains a paucity of data on the role of telomerase and its regulation in atherosclerosis. The increased prevalence of atherosclerosis in older people is associated with a decline in the function of lymphoid progenitors and mature T-cells,^34^ and shorter telomeres have been observed in leukocytes of human patients with advanced coronary atherosclerosis.^35^ However, it is unclear as to whether accelerated telomere shortening or inherited telomere length are the most important influences in the development of atherosclerosis.^36,37^

It has been previously suggested that telomere shortening or dysfunction in telomerase activity in leukocytes, including T-cells, may be causative in the progression of this disease.^29,31^ This hypothesis, however, is in direct contradiction to the recent demonstration of Poch et al^38^ who have shown that a mouse model that lacks telomerase activity and displays significantly shorter telomeres is in fact protected from atherosclerosis,^38^ as well as the observations that aged rabbits develop less atheroma under a high-cholesterol diet compared with younger animals.^39^ Several human studies have also failed to identify any association between telomere length and atherosclerosis^40^ or between telomere length and mortality in subjects over the age of 65 years.^41–43^ However, it has been suggested that studies showing no correlation have examined older populations^37^ while until the age of 60 years there is a correlation between telomere length and mortality which includes cardiovascular deaths.^44^ Adding further complexity to the interpretation of the human studies is the fact that factors, including race/ethnicity and sex, all influence outcome.^37^

In this study, we aimed to address some of the controversy surrounding the role of telomerase activity and telomere length with regards to T-cell activation and proliferation. Moreover, we aimed to address the functional requirement of telomerase for regulatory T-cell–mediated atheroprotection. We report here that oxidative stress suppresses telomerase activity and attenuates proliferation of CD4^+^ T-lymphocytes but not CD11b^+^ myeloid cell expansion. Lack of *Tert* in cells with sufficiently long telomeres within a population of T_reg_ T-lymphocytes is not detrimental to their suppressive function. In contrast, short telomeres diminished T_reg_ number and function.

## Methods

The data that support the findings of this study are available from the corresponding author on reasonable request. Details of the major resources and detailed methods can be found in the online-only Data Supplement.

### Animals and Ethics

Animal work was authorized and approved by the Cambridge and Newcastle University Ethics review boards. All animal procedures were performed conforming to the guidelines from Directive 2010/63/EU of the European Parliament on the protection of animals used for scientific purposes. Both male and female mice were used in all studies. TERT knockout, generated by Chiang et al^45^ (Jax strain B6.129S-Tert tm1Yjc/J), and TERC knockout, generated by Blasco et al^46^ (Jax strain B6.Cg-Terc tm1Rdp/J), animals were purchased from Jackson Laboratory, Maine. Generation and initial phenotypic characterization of the *mTert*-GFP (green fluorescent protein) mice have been published previously.^47–51^ The *mTert*-GFP mouse^2^ contains a reporter cassette in which the expression of the gene for GFP is under the control of a 4.4-kb fragment of the promoter of murine *Tert.* As such GFP expression in this model represents *mTert* promoter activity as an indicator of TERT transcription. Rag2^−/−^ ApoE^−/−^ (recombination activating gene 2/apolipoprotein E) double knockout mice and CD28^−/−^ mice were originally obtained from Charles River. All mice were held under the UK Home office animal licenses PPL 60/3864 or PO11C464C. Details for each line used to obtain the data for each figure are included in Table I in the online-only Data Supplement.

### Splenocyte and CD4 Cell Isolation, Culture, and Growth Curves

Cells were isolated and cultured as described previously.^47^ Assessment of CD4^+^ cell purity is demonstrated in Figure I in the online-only Data Supplement. Splenocytes were cultured in a 24-well plate (2×10^6^ cells/2 mL per well). MACSibead mouse T-cell, CD3 and CD28 antibody coated, expansion beads (Miltenyi 130-093-627) were added to medium as described.^47^ TA-65 activator (TA65) is a telomerase activator purified from Astragalus membranaceous^52^ and provided by TA-Science Inc (New York, NY). BIBR 1532 (Tocris Bioscience), a telomerase inhibitor,^53^ was dissolved in dimethyl sulfoxide and used as the indicated concentration.

### Dihydroethidium and Mitosox Staining

Dihydroethidium and Mitosox are established methods to measure superoxide levels.^54,55^ Cells were labelled with 10-μM dihydroethdium (Molecular Probes) as described^56^ or 5-μM Mitosox Red (Molecular Probes).

### Telomeric Repeat Amplification Protocol Polymerase Chain Reaction ELISA

Telomeric Repeat Amplification Protocol kit (Roche) was performed as per the manufacturer’s instructions. TERT^−/−^ splenocytes and the immortal fibroblast cell line 3T3 were used as negative and positive controls (Figure VI in the online-only Data Supplement).

### Detection of T_reg_

After isolation, splenocytes were labeled using the T_reg_ Detection Kit (Miltenyi Biotec, Auburn, CA) as per manufactures instructions. In our hands, ≥98% of CD4+ T-cells can be identified as T-cells by CD3^+^ staining (Figure V in the online-only Data Supplement).

### Atherosclerosis Experiments

Rag2^−/−^ ApoE^−/−^ mice were transplanted with 10^7^ splenocytes from CD28^−/−^ mice and either PBS or 10^6^ CD4^+^ CD25^+^ regulatory T-cells from either Tert^−/−^ mice or wild-type (WT) littermates. Mice were fed an atherogenic Western diet (21% fat, 0.15% cholesterol) for 7 weeks. Atherosclerosis was quantified in the aortic root as described previously.^57^

### Statistical Analysis

After a test for normality, statistical analysis was performed as appropriate and indicated in the legend of each figure. Data are presented as mean±SEM or as dot for individual experiments with a line representing the median. A Mann–Whitney *U* test was used to compare groups of 2, and 2-way ANOVA with Bonferroni post hoc analysis was used to compare groups of ≥3. Statistical significance was set at *P*<0.05.

## Results

### Increased Oxidative Stress Results in Chronic T-Cell Activation and Reduced Proliferation

Increased oxidative stress and acute inflammation as well as increased T-cell activity are key characteristics of atherosclerosis. We have previously established hyperoxia as a model of chronic mild oxidative stress.^18,58,59^ In this study, we used hyperoxic culture conditions to study the effect of chronic oxidative stress on lymphocyte growth kinetics and T-cell activation.

**Figure 1.**
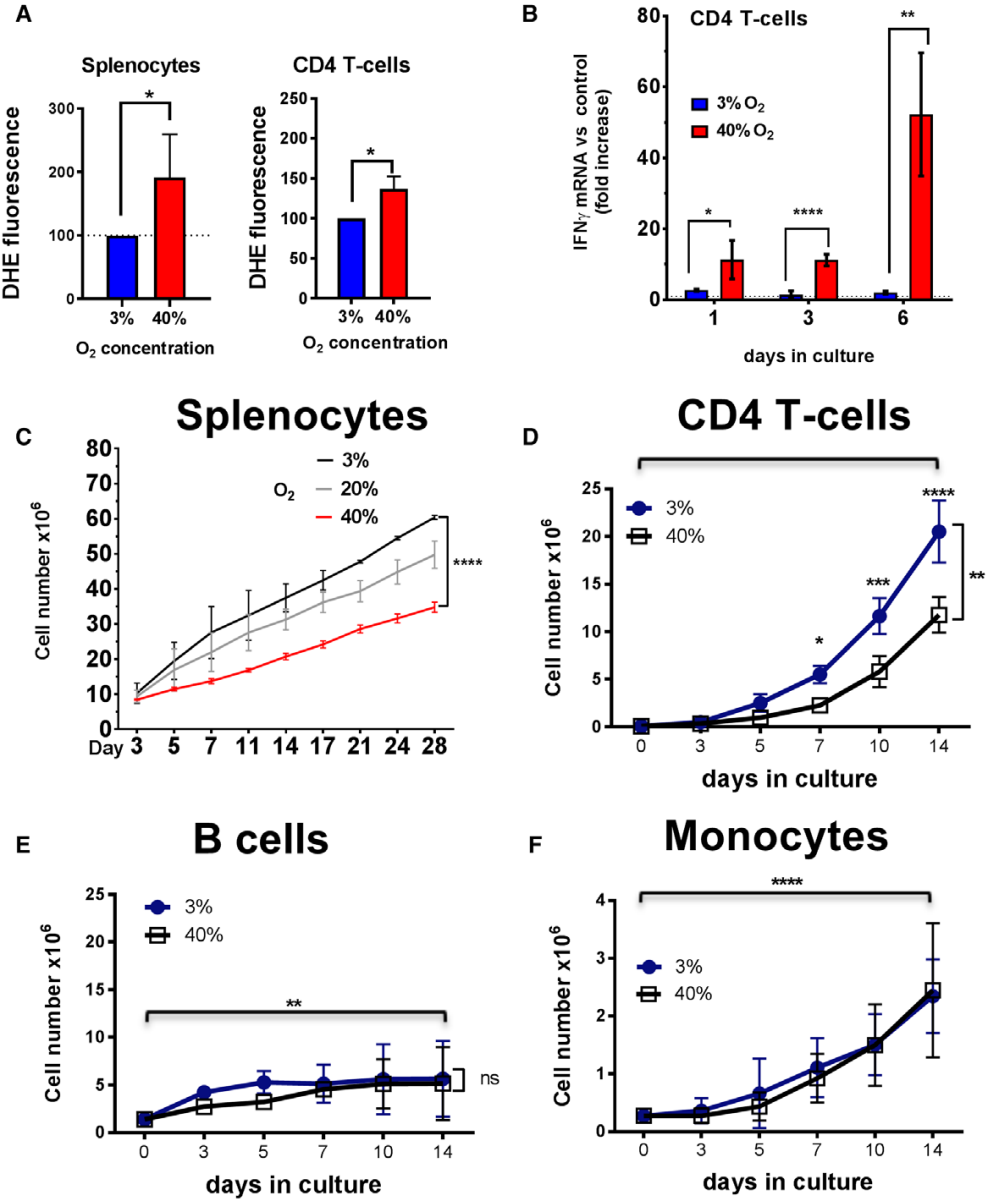
Oxidative stress leads to chronic interferon γ (INF-γ) expression and impairs proliferation of CD4 T-cell but not B-cells or myeloid cells. **A**, Total or CD4^+^ splenocytes were cultured at either 3% or 40% oxygen saturation for 10 d. Dihydroethidium (DHE) fluorescence was increased under hyperoxia. n=4 for each experimental group. **B**, CD4^+^ splenocytes were cultured for up to 6 d at either 3% or 40% oxygen saturation. IFN-γ transcript expression was significantly increased at all time points under hyperoxia as quantified by quantitative reverse transcription polymerase chain reaction. n=4 for each experimental group. **C**, 2×10^5^ splenocytes were cultured per well at 3% or 40% oxygen saturation. Total cell numbers were quantified at 3-d intervals. All error bars represent the standard deviation (SD). n=4 for each experimental group. **D** and **F**, Flow cytometry was used to identify and quantify individual cellular subpopulations of the splenocyte cell cultures. A total of 2×10^6^ splenocytes/mL were cultured after T-cell–specific activation. Total numbers of each subpopulation were quantified by flow cytometry. n=3 for each experimental group, error bars (SD). *P<0.05, **P<0.01, ***P<0.001, and ****P<0.0001 using with Mann–Whitney *U* test or 2-way ANOVA as appropriate.

Splenocytes or CD4^+^ T-cells were cultured for 10 days at either physiological oxygen levels (physoxia, 3% oxygen) or hyperoxia (40% oxygen). Assessment of cell purity is shown in Figure I in the online-only Data Supplement. When cultured under hyperoxic conditions, both the total splenocyte population and CD4^+^ T-cells displayed a significantly higher level of dihydroethidium staining intensity indicating increased mitochondrial superoxide levels (Figure 1A). Similar results for splenocytes cultured in high oxygen were obtained with another superoxide-specific probe Mitosox (Figure II in the online-only Data Supplement).

CD4^+^ cells cultured under hyperoxic conditions had an elevated interferon γ mRNA expression relative to splenoctyes cultured at physoxia (Figure 1B). Increased interferon γ expression was observed throughout the culture duration, indicative of chronic T-cell activation, with a further increase at 6 days. High oxygen had a similar effect on the expression of interleukin 2 (Figure III in the online-only Data Supplement). Having demonstrated that hyperoxia and oxidative stress are associated with aspects of T-cell activation, we were interested to examine the effect of oxidative stress on splenocyte proliferation. Splenocytes cultured at physoxia demonstrated a 30-fold increase in cell number over 28 days. In contrast, splenocytes cultured in either 20% or 40% oxygen saturation showed a dose-dependent attenuation of proliferation by 15% or 50%, respectively, compared with physoxia (*P*<0.0001; Figure 1C).

Given the heterogeneity of cell populations residing in the spleen, flow cytometry was used to study the effect of oxidative stress on the proliferation of individual splenocyte subpopulations (Figure 1D–1F). Gating strategy and controls shown in Figure IVA and IVB in the online-only Data Supplement. As CD4 is also expressed on other lymphocyte subpopulations, including dendritic cells, we first quantified what percentage of CD3^+^ T-cells is contained within the CD4^+^ population. Over 98% of CD4^+^ cells also expressed CD3 (Figure V in the online-only Data Supplement). To reflect the physiological environment, the total splenocyte population was cocultured in the presence of T-cell–specific activation under conditions of physoxia or hyperoxia. After 14 days, total cell numbers of the individual subpopulations were quantified by flow cytometry to extrapolate rates of proliferation. CD4^+^ T-cells displayed the greatest proliferative response with 100-fold expansion for 14 days under 3% oxygen (Figure 1D) while a 3-fold increase in B-cell numbers (Figure 1E) and a 7-fold increase in the CD11b^+^ population (including myeloid cells, granulocytes, and natural killer T-cells; Figure 1F) was observed during the same period. Importantly, only the proliferation of CD4^+^ T-cells was significantly (*P*<0.01) attenuated by hyperoxia-induced oxidative stress. However, the CD11b^+^ were not experimentally stimulated to proliferate under these experimental conditions.

### Oxidative Stress Impairs T-Cell Proliferation via Suppression of Telomerase

**Figure 2.**
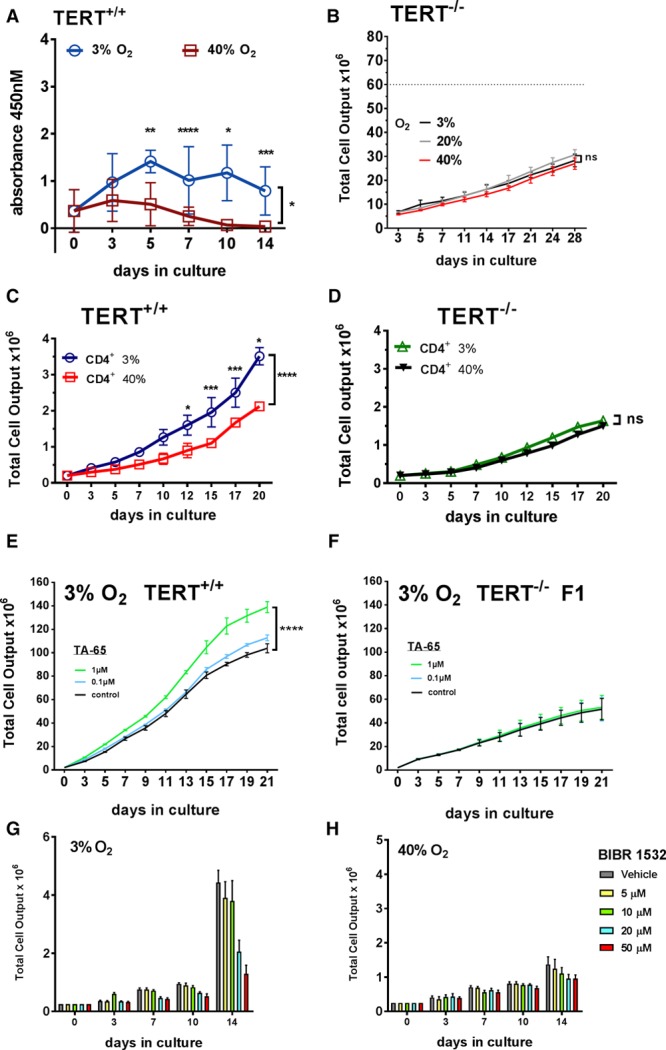
Oxidative stress decreases T-cell proliferation and suppresses telomerase. **A**, 2×10^6^ splenocytes (n=5) were cultured for up to 14 d at either 3% or 40% oxygen. T-cell–specific activation was achieved by coating plates with CD3 and CD28 antibodies. Telomeric Repeat Amplification Protocol assay was used to quantify telomerase activity in cells isolated at the 3, 5, 7, 10, and 14 d time points. n=4 for each experimental group. **B**, Splenocytes isolated from telomerase reverse transcriptase (TERT)^−/−^ mice were cultured for up to 27 d at either 3%, 20%, or 40% oxygen saturation, and total cell numbers were quantified at 2- to 4-d intervals. Dotted line indicates mean cell numbers for wild-type splenocytes after 28 d of cultivation at the same oxygen saturation. n=4 for each experimental group. **C** and **D**, CD4^+^ splenocytes from either TERT^+/+^ or TERT^−/−^ mice were cultured at 2×10^5^ cells/well under 3% or at 40% oxygen conditions for 14 d. T-cell–specific activation was maintained by addition of antibody-labeled beads at days 0, 7 and 14. n=3 for each experimental group. **E** and **F**, Total splenocytes from either TERT^+/+^ or TERT^−/−^ mice were cultured at 1×10^6^ cells/well under 3% in the presence of the telomerase activator TA-65 at indicated concentrations for up to 21 d. T-cell–specific activation was maintained by antibody-coated plates. n=3 for each experimental group. **G**, CD4^+^ splenocytes were cultured at 2×10^5^ cells/well under 3% n=3 for each experimental group, or at (**H**) 40% oxygen conditions for 14 d n=4 for each experimental group. In the presence of BIBR 1532 (telomerase inhibitor) at indicated concentrations, T-cell–specific activation was maintained by addition of antibody-labeled beads (anti-CD3/CD28) at days 0, 7 and 13. All error bars represent the SD. *P<0.05, ****P<0.0001 using with Mann–Whitney *U* test or 2-way ANOVA as appropriate. ns indicates nonsignificant.

We next sought to identify the underlying mechanism by which hyperoxia attenuates T-cell proliferation. We have previously demonstrated that increased oxidative stress excludes the TERT protein from the nucleus of hTERT (human telomerase reverse transcriptase) overexpressing fibroblasts, thereby preventing telomere maintenance and halting cell proliferation^60^ and that telomerase facilitates statin-induced T-cell proliferation.^47^ The latter led us to hypothesize that the oxidative stress–induced attenuation of T-cell proliferation in this study might be mediated via the suppression of telomerase activity. To test this hypothesis, we quantified telomerase activity in WT splenocytes cultured at either physoxia or hyperoxia. Hyperoxia resulted in a significant reduction of telomerase activity, and all detectable telomerase activity was suppressed after 2 weeks of culture (*P*<0.0001; Figure 2A). Telomeric Repeat Amplification Protocol assay positive and negative controls are shown in Figure VI in the online-only Data Supplement.

To specifically address the role of telomerase in mediating proliferation, we investigated the proliferative potential of splenocytes isolated from first generation (F1) TERT knockout mice, which lack expression of telomerase reverse transcriptase (Tert^−/−^), the catalytic subunit of telomerase.^61^ Although these mice lack functional telomerase activity, they do not have significantly shorter telomeres than WT animals.^47^ No significant difference in the rates of proliferation in Tert^−/−^ splenocytes cultured under physoxic or hyperoxic conditions were observed (Figure 2B). Moreover, CD4^+^ T-cells immunomagnetically isolated from spleens of F1 Tert^−/−^ mice, opposed to WT CD4^+^ cells (Figure 2C), also displayed no difference in proliferation rates when cultured in either hyperoxia or physoxia (Figure 2D). To further validate our findings, WT splenocytes were cultured in the presence of the small molecule telomerase activator TA-65.^52^ Activation of telomerase in cultured WT splenocytes significantly enhances proliferation in a dose-dependent manner (Figure 2E). As expected, even in the absence of TA-65, TERT^−/−^ splenocytes demonstrate a decreased baseline proliferation compared with WT cells (Figure 2E and 2F) and failed to respond to TA-65 (Figure 2F). Finally, we investigated the effects of the specific telomerase inhibitor BIBR-1532^53^ on splenocyte proliferation at different oxygen saturation levels. Splenocytes demonstrated a dose-dependent attenuation of proliferation when treated with the inhibitor providing further evidence that telomerase activity seems to mediate proliferation when cultured at physoxia (Figure 2G). As expected, splenocytes cultured in hyperoxia demonstrated a reduced proliferation; however, BIBR 1532 treatment resulted in a significant dose-dependent decrease in proliferation at 2 weeks even under these conditions (Figure 2H).

**Figure 3.**
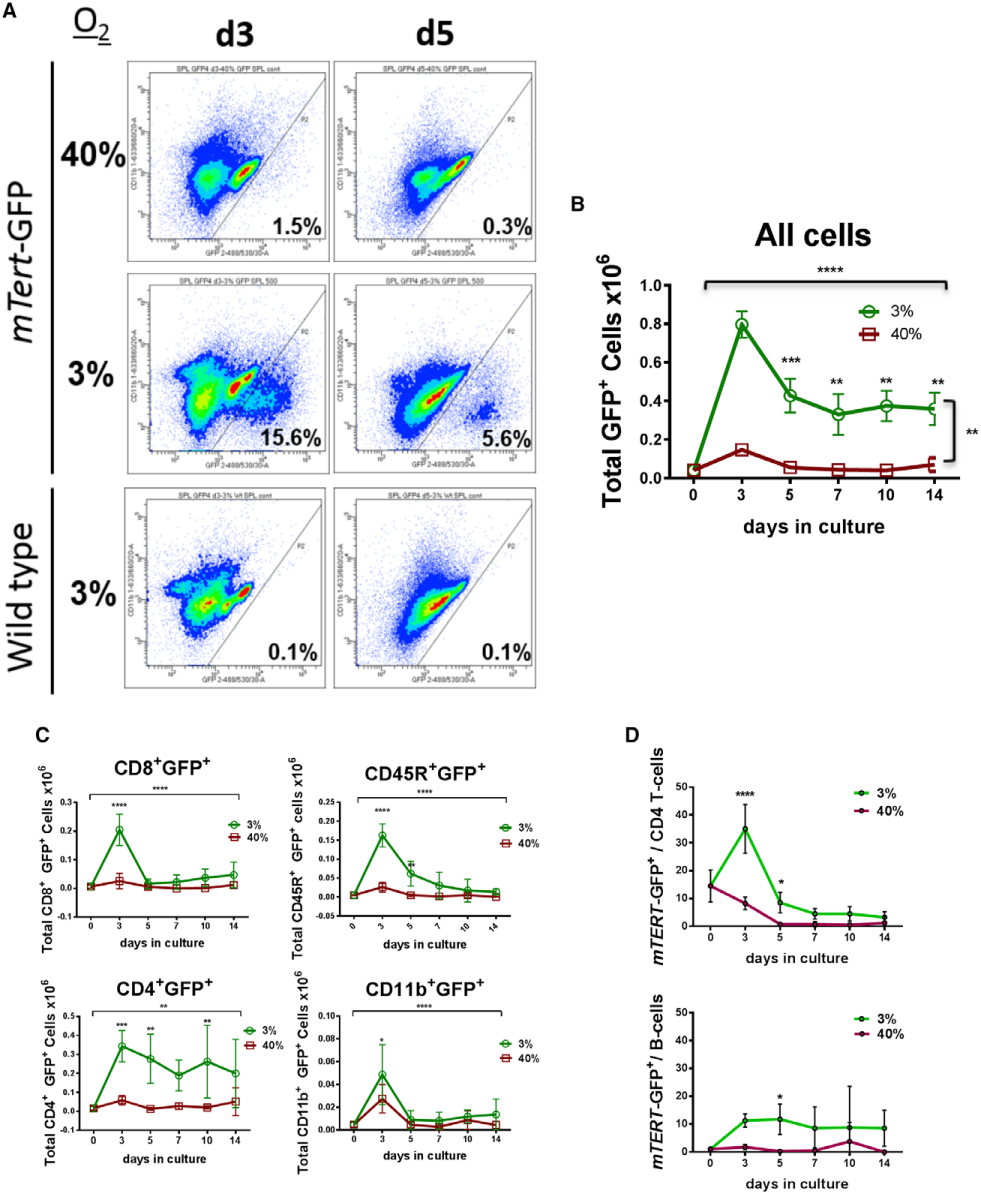
Oxidative stress suppresses telomerase at the level of mTert transcription. Splenocytes were isolated from *mTert*-GFP reporter mice and cultured for 14 d at 3% or 40%, and T-cell activation maintained on antibody-coated plates. *mTert*-GFP (green fluorescent protein) expression was quantified at a single cell level on 3, 4, 7, 10, and 14 d of cultivation by flow cytometry. **A**, Representative flow dot plots for the percentage of total *mTert*-GFP expressing cells at the indicated time points and at both conditions as a percentage of total cells. Gating was established using wild-type mice as controls with <0.1% positive events in the *mTert*-GFP^+^ gate. **B**, Absolute numbers of *mTert*-GFP–positive cells within the total splenocyte population over time as quantified by flow cytometry. n=4 for each experimental group. **C**, Absolute numbers of *mTert*-GFP–positive cells within individual splenocyte subpopulations over time as quantified by flow cytometry. n=4 for each experimental group. **D**, Percentage of the CD4 (top graph) and B-cell populations (bottom graph) that express *mTert*-GFP at each time point and condition. n=3 for each experimental group. All error bars represent the SD. *P<0.05, **P<0.01, ***P<0.001, and ****P<0.0001 using 2-way ANOVA.

Having demonstrated that oxidative stress suppresses telomerase activity, we next aimed to ascertain whether this reduction in telomerase was at a transcriptional level of *TERT* expression. To investigate the effect of oxidative stress on *mTERT* expression at the single cell level, splenocytes were isolated from telomerase reverse transcriptase reporter mice (*mTert*-GFP) and cultured under the different oxygen conditions (Figure 3A). Under physoxia, ≈15% of splenocytes expressed *mTert*-GFP at day 3 and expression decreased thereafter during ongoing cultivation (Figure VIIA and VIIB in the online-only Data Supplement). In contrast, hyperoxia significantly (*P*<0.0001) attenuated *mTert*-GFP expression in splenocytes at all time points investigated (Figure 3B) demonstrating that oxidative stress inhibits *mTert* expression at a transcriptional level. We next evaluated the total number of *mTert*-GFP expressing cells and the percentage of *mTert*-GFP expressing cells in individual splenocyte subpopulations (Figure 3C and 3D; gating strategy is shown in Figure IV in the online-only Data Supplement). *mTert*-GFP expression mimicked the proliferative trends of each cell subpopulation in the different oxygen conditions shown in Figure 2.

### Telomerase-Deficient T_reg_ Cells With Long Telomeres Remain Protective Against Atherosclerosis

**Figure 4.**
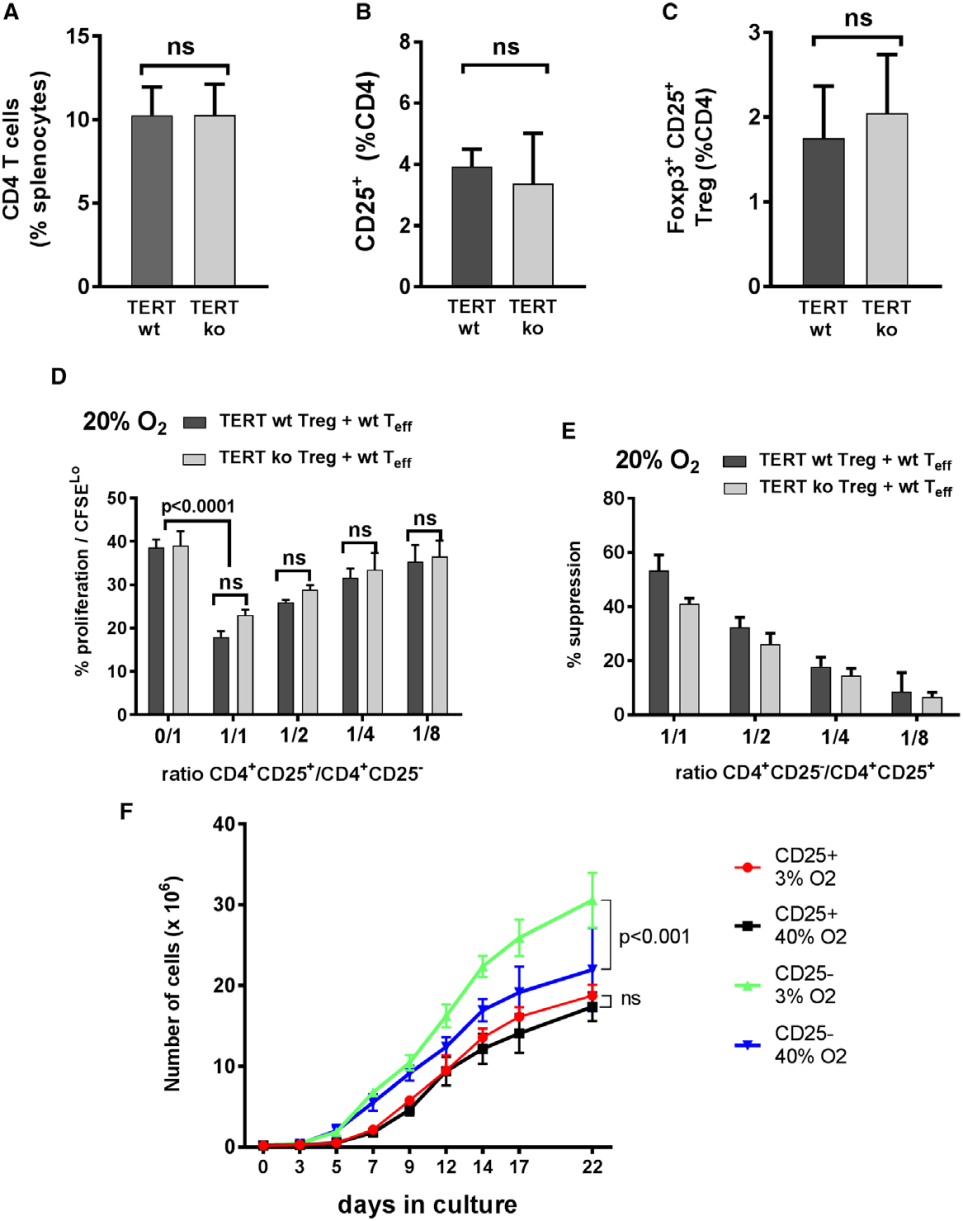
Absence of telomerase reverse transcriptase does not affect the in vivo number or suppressive function of regulatory T-cell (Treg) cells in vitro. **A**–**C**, Flow cytometry was used to quantify CD4^+^ and T_reg_ cell populations in the spleens of wild-type (WT) or telomerase reverse transcriptase (TERT)^−/−^ mice. n>4 for each experimental condition. **D** and **E**, CD4^+^CD25^−^ effector T-cells (T_eff_) cells were isolated and carboxyfluorescein diacetate succinimidyl ester (CFSE) labeled to allow quantification of proliferation by flow cytometry (CFSE is diluted during each cell division). Coculture with either WT or TERT^−/−^ T_reg_ cells suppressed T_eff_ cell proliferation in a dose-dependent manner. n>5 for each experimental condition. **F**, CD25^+/−^ cells were isolated from the spleen of WT mice and were cultured for up to 27 d at either 3% or 40% oxygen saturation, and total cell numbers were quantified at indicated intervals. n=3 for each experimental condition. Error bars represent SD. A 2-way ANOVA was used for statistical analysis.

We have previously established that T_reg_ cells modulate the development of atherosclerosis in mice. Using adoptive transfer studies, we demonstrated that naturally arising T_reg_ cells are potent inhibitors of atherosclerosis in several different mouse models.^5^ We next examined whether telomerase-deficient T_regs_ retain the potential to suppress atherosclerosis in vivo. Surprisingly, Tert^−/−^ mice contained the same number of both CD4^+^ T-cells and T_reg_ cells, including the CD4^+^CD25^+^FoxP3^+^ population, as WT mice (Figure 4A–4C). Gating strategy and controls shown in Figure VIIC in the online-only Data Supplement. While acknowledging the potential variation introduced by the digestion protocol, we also quantified the total number of splenocytes obtained from each spleen for WT and TERT^−/−^ mice. A significant reduction in the total number of cells isolated from TERT^−/−^ was observed (Figure VIII in the online-only Data Supplement). To evaluate the functional properties of telomerase-deficient T-cells, we purified T_reg_ cells and CD4^+^CD25^–^ effector T-cells and assessed their proliferative potential ex vivo (representative flow plots are shown in Figure IX in the online-only Data Supplement). T_reg_ cells were suppressive of effector T-cells proliferation, regardless of Tert expression (Figure 4D and 4E). Having identified that telomerase expression was not required for T_reg_ suppressive function ex vivo, we investigated the effect of oxidative stress specifically in this population in vitro (Figure 4F). Hyperoxia had no effect on the expansion of the CD25^+^ T_reg_ population but did significantly diminish proliferation of CD25^–^ effector T-cells.

**Figure 5.**
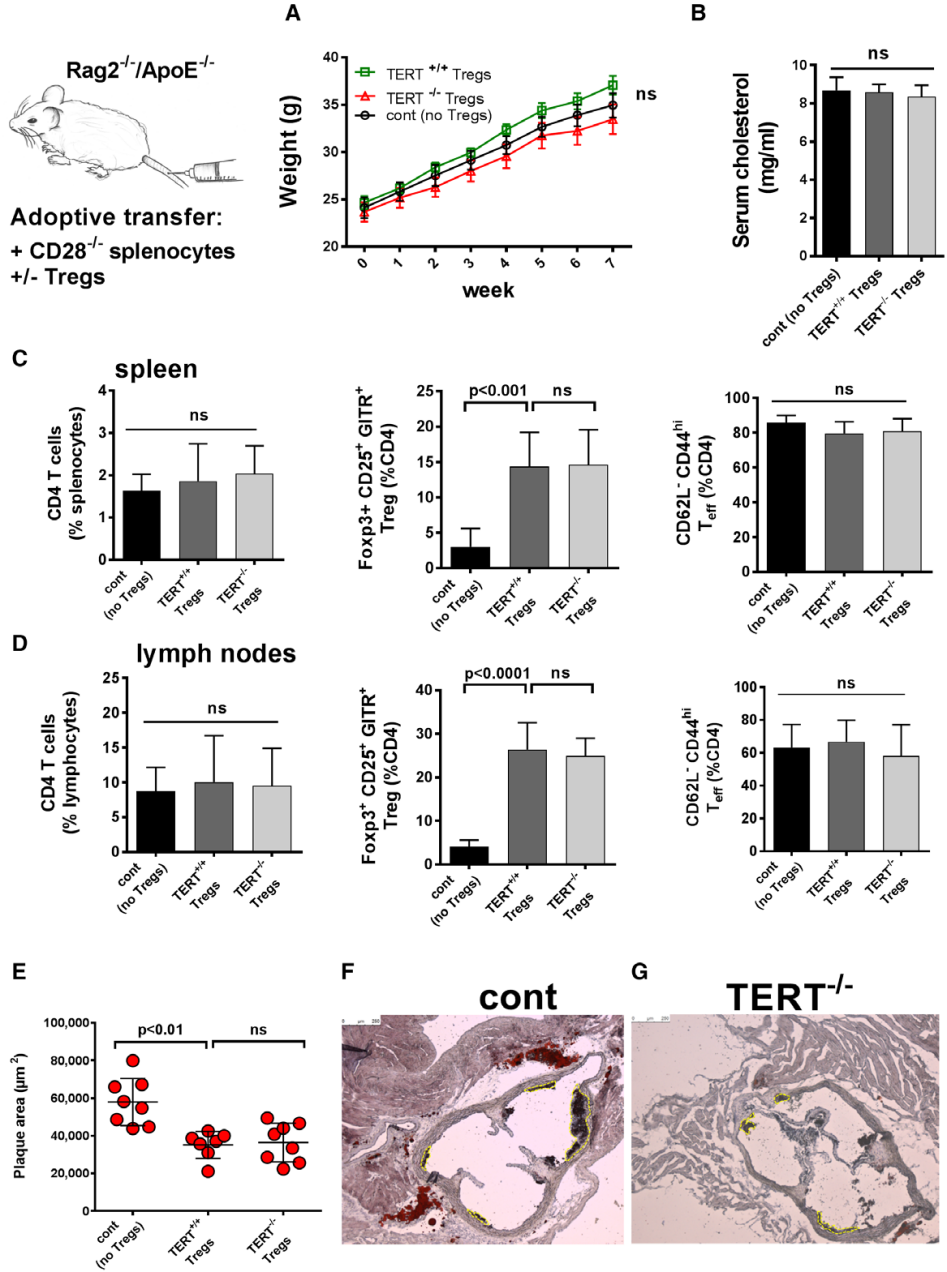
Regulatory T-cell (T_reg_) cells lacking telomerase reverse transcriptase remain protective against atherosclerosis. **A**, Rag2^–/–^ (recombination activating gene 2) mice were transplanted with telomerase reverse transcriptase (TERT)^−/−^, wild-type (WT) T_regs_, or no T_regs_ (control) and weighed daily. No significant difference in weight was observed between any groups. **B**, Measurements of serum cholesterol. No significant difference was observed between groups. **C** and **D**, No difference in the number of viable CD4^+^ or T_reg_ was observed within the lymph node and spleens of mice transferred with either WT or TERT^−/−^ T_regs_. Quantification performed by flow cytometry. **E**, Comparison of plaque area between Rag2^–/–^ mice transplanted with WT T_reg_, TERT^−/−^ T_regs_, or no T_regs_. **F** and **G**, Representative images of plaques stained with Oil Red O in mice transplanted with TERT^−/−^ T_regs_ or no T_regs_. Yellow borders highlight plaque area. The data were obtained from 9 mice for each experimental group. Error bars represent SD. A Mann–Whitney U test or 2-way ANOVA was used for statistical analysis as appropriate.

To ascertain whether TERT-deficient T_reg_ cells remain protective against atherosclerosis in vivo, we adoptively transferred a mixture of CD28^−/−^ splenocytes, which lack the potential to differentiate into T_reg_ cells, together with either WT or Tert^−/−^ T_regs_ into Rag2^−/−^ApoE^−/−^ mice, which lack mature lymphocytes. No group of atherogenic Western diet–fed mice displayed any difference in their weight (Figure 5A) and had the same elevated levels of cholesterol (Figure 5B). As expected, total and effector CD4^+^ T-cells among splenocytes were the same in all 3 groups (Figure 5C) while T_regs_ were significantly higher in the adoptive transfer groups (15% versus 3%; *P*<0.001; Figure 5C and 5D). The same was true for T_reg_ content in lymph nodes (25% versus 4%; *P*<0.001; Figure 5D). For all subpopulation analyses, gating strategy and representative flow plots are shown in Figure XA and XB in the online-only Data Supplement. We assessed the atherosclerotic plaque area in the aorta after 7 weeks of Western diet feeding. TERT^−/−^ T_regs_ were able to suppress plaque area to the same degree as WT T_regs_ (*P*<0.01; Figure 5E–5G).

### Telomere Shortening Leads to Reduced Numbers of T_regs_ and Decreases T_reg_ Cell Function

**Figure 6.**
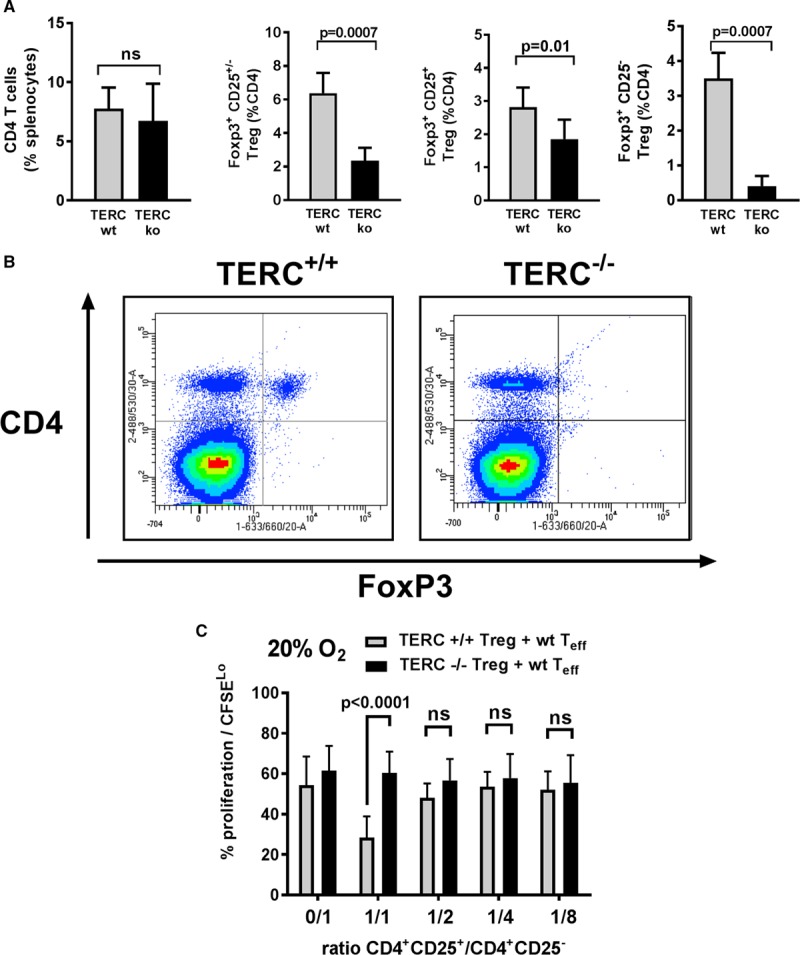
Telomerase RNA component (TERC)^−/−^ mice have reduced regulatory T-cell (T_reg_) numbers with decreased suppressive function ex vivo. **A**, Flow cytometry was used to quantify CD4^+^ and T_reg_ cell populations in the spleens of wild-type (WT) or TERC^−/−^ mice. n>5 for each experimental condition. **B**, Representative example of flow cytometry plot. **C**, CD4^+^CD25^–^ effector T-cells (T_eff_) were isolated and carboxyfluorescein diacetate succinimidyl ester labeled to enable quantification of proliferation by flow cytometry. n=9 for each experimental condition. Coculture with WT (C57BL/6J) but not TERC^−/−^ T_reg_ cells suppressed T_eff_ cell proliferation in a dose-dependent manner. Error bars represent SD. A Mann–Whitney *U* test was used for statistical analysis.

We next aimed to ascertain whether telomere attrition in the T_reg_ cell population affects their number and function. We have previously demonstrated that bone marrow hematopoietic stem and progenitor cells from 12-month-old first generation (F1) TERC^−/−^ mice had shorter telomeres compared with WT mice from the same strain (because the heterozygous used to generate TERT^−/−^ mice already having short telomeres).^62^ We compared the percentage of CD4^+^ T-cells and T_regs_ in the spleen of both these TERC^−/−^ and WT mice (Figure 6A and 6B). Genetic knockout of TERC did not affect CD4^+^ T-cell numbers; however, it did result in a significant reduction in the number of T_regs_ (CD4^+^CD25^+^Foxp3^+^; Figure 6B). In addition, we assessed the functional properties of TERC-deficient T_regs_ ex vivo. Surprisingly, unlike TERT-deficient cells (Figure 4D and 4E), TERC^−/−^ T_reg_ cells lacked suppressive function of effector T-cells proliferation (Figure 6C).

## Discussion

Our data demonstrate that telomerase positively influences T-cell expansion; however, it is not required per se for baseline proliferation or T-cell homeostasis. Evidence suggests that activated T-cells express high levels of telomerase activity to protect their telomeres from accelerated shortening, thereby evading replicative senescence.^63^ However, the contribution of telomerase activity to T_reg_ function had not been investigated to date.

The data we present in this study indicate that telomerase expression is not required for T_reg_ suppressive function. Although absolute numbers of splenocytes may be decreased, mice with long telomeres that lack functional telomerase activity, like the F1 TERT^−/−^ mice used in this study, maintain a T_reg_ population of relative comparable size to WT animals, which remains functional. Although T_regs_ express telomerase, it is possible that this cannot indefinitely maintain telomere length, particularly under conditions of oxidative stress that decreases telomerase activity. Interestingly, we have demonstrated that TERC^−/−^ mice with shorter haematopoietic stem and progenitor cell telomere length have a severe reduction in peripheral T_reg_ cell numbers in vivo and impaired function in vitro. This confirms that indeed it is a sufficiently long telomere length rather than the mere presence of telomerase activity that is important for T_reg_ cell functioning.

For our studies, we have performed adoptive transfer experiments using Rag2^−/−^ ApoE^−/−^ mice, as previously described.^5^ These experiments require the transfer of CD28^−/−^ splenocytes which have the potential to differentiate into all lineages of the adaptive immune system except T_reg_ cells. We are aware that although our study directly tested the functionality of TERT^−/−^ T_reg_ cells in an in vivo disease model, the immune system may be compromised in this model. Further studies are required to determine whether more subtle phenotypes are apparent in alternative nonimmunocompromised models that lack TERT.

Multiple studies have confirmed the association of short leukocyte telomere length and the incidence of CHD in humans.^64^ Mendelian randomization studies in >200 000 participants have also suggested that a genetic reduction of telomere length (via mutations in TERT or TERC genes) leads to an increased risk of CHD.^65^ Although in our animal studies absence of telomerase had no effect on T_reg_ maintenance, it is important to point out that because of the long telomeres in laboratory mouse strains, this may not reflect the human biology of telomeres. Indeed, late-generation TERC knockout mice with shorter telomeres are considered more representative of the human situation.

It is known that the adaptive immune system plays a pivotal role in the development and progression of atherosclerosis.^66^ Although the proatherogenic function of T-helper type 1 CD4^+^ as well as CD8^+^ T-cells is well established, it is also emerging that T_reg_ cells are critical in offsetting the detrimental effects of both adaptive and innate immune responses. What remains unclear is how the plaque environmental milieu influences the net effect of these 2 antagonistic responses and how the molecular mechanisms controlling the survival, proliferation, and suppressive function of the T_reg_ population contribute to disease progression. We propose that within the atherosclerotic plaque, chronic oxidative stress conditions may lead to suppression of telomerase and acceleration of telomere attrition in T_regs_. This in turn may contribute to premature senescence of T_reg_ cells and therefore progression of atherosclerosis.

Oxidative stress within the atherosclerosis microenvironment may have other detrimental effects that could contribute to disease progression. Oxidative stress can directly damage both genomic DNA, in the absence of telomere shortening, and mitochondrial DNA, which is linked to vascular senescence and atherosclerosis.^67^ Accumulated DNA damage in peripheral blood cells not only occurs in patients with coronary artery disease and acute myocardial infarction^68^ but is also associated with outcome.^69^ Moreover, high-cholesterol diet and atherosclerosis have been associated with increased DNA damage in peripheral lymphocytes in rabbits.^70^ Mitochondrial localization of the TERT protein has been demonstrated to regulate levels of mitochondrial derived reactive oxygen species and TERT can translocate to the mitochondria reducing mitochondrial derived reactive oxygen species production in the process, which is independent of its canonical role in the nucleus.^60^ It had been shown in different cell types that lack of TERT specifically in the mitochondria is detrimental to mitochondrial function and morphology.^71^ However, such a mitochondrial role has to date not been described in immune cells. We and others have previously demonstrated that TERT can to bind to mitochondrial DNA which also protects cells from DNA damage and oxidative stress.^71,72^ An antioxidant role of TERT has also recently been described by Beyer et al^53^ who have demonstrated that loss of telomerase activity in cells of healthy human vessels results in a switch from nitrous oxide to a proinflammatory hydrogen peroxide, which mediates vascular dilation. Conversely, restoration of telomerase activity in arterioles from humans with coronary artery disease reverts the mediator of flow-induced dilation from hydrogen peroxide to nitrous oxide.^53^ The data in our current study demonstrate that oxidative stress attenuates TERT expression at a transcriptional level in lymphocytes. Such reduction in telomerase influences both telomerase nuclear activity and would also diminish some noncanonical functions of telomerase and thereby resistance to oxidative stress further contributing to disease. Indeed, we have demonstrated that oxidative stress and attenuated TERT expression are both associated with an increased production of mitochondrial derived superoxide. We are currently investigating the impact of telomerase activation by TA-65 on canonical (telomere length) and noncanonical (oxidative stress and microvascular endothelial function) pathways in patients with myocardial infarction (TACTIC trial [Telomerase Activator to Reverse Immunosenescence in Acute Coronary Syndrome: A Double-Blind, Phase II, Randomised Controlled Trial]). Noncanonical roles for telomerase may possibly also explain the difference in phenotype between the TERT^−/−^ and TERC^−/−^ mice in addition to differences in telomere length that are already differentially regulated at the level of heterozygotes in both knockout models.^73^ The TERC^−/−^ mice might still benefit from noncanonical roles of telomerase because of the presence of TERT.

Correlative evidence from human population studies collectively suggests an association of short telomeres in lymphocytes with conditions of increased oxidative stress, including smoking, obesity, and CHD.^27,28,32,33,74–76^ Furthermore, nonimmune cells have been shown to have accelerated telomere attrition and develop premature senescence when cultured under hyperoxic conditions.^18,47^ This could represent one mechanism that would explain why patients with short telomeres in lymphocytes are predisposed to CHD, a hypothesis supported by our recent work demonstrating that immunosenescence can be an independent predictor of disease outcome in older people, possibly via accelerated atherosclerosis.^77^

The only study showing an atheroprotective effect of global telomerase deficiency as well as shortened telomeres in the ApoE mouse model^38^ may be explained by global immunosenescence and a dysfunctional immune system, where the lack of myeloid cell proliferation is responsible for reduced plaque progression. Of note, our experiments demonstrate that in contrast to T-cells, proliferation of myeloid cells was indeed not influenced by oxidative stress. Therefore, the plaque environment seems to modulate the local immune response in a manner conducive for chronic inflammation. A dichotomous protective versus pathological role of telomerase as a result of tissue specificity has already been described (reviewed in 78). Telomerase activation has been demonstrated to reduce reactive oxygen species production and thereby inflammation in the endothelium whereas increased telomerase activity within the vascular smooth muscle layer can result in abnormal proliferation and vascular remodeling in hypertensive rats.^78^

Finally, even in light of recent studies demonstrating that critically short telomeres can be protective for atherosclerosis, our current data suggest that restoring telomerase activity in T-cells, for example, through small molecule activators such as TA-65,^52,79^ remains a potentially powerful therapeutic intervention.

## Acknowledgments

We acknowledge the Newcastle University Flow Cytometry Core Facility for assistance with the generation of Flow Cytometry data.

## Sources of Funding

This study was supported, in part, by British Heart Foundation Project Grants PG/15/85/31744 and PG/12/47/29681 (www.BHF.org.uk) as well as the Newcastle Healthcare Charity (www.newcastle-hospitals.org.uk/patient-guides/charity-matters-at-newcastle-hospitals_charitable-funds.aspx). N.M. Al Zhrany was funded by a stipend from the Government of Saudi Arabia.

## Disclosures

None.

## Supplementary Material

## Notes

Highlights

- Hyperoxia induces oxidative stress and T-cell activation and reduced CD4^+^ T-cell proliferation in vitro.
- Oxidative stress–mediated reduction in CD4^+^ T-cell proliferation is telomerase reverse transcriptase dependent.
- Telomerase reverse transcriptase deficiency has no effect on regulatory T-cell (T_reg_) numbers in vivo or suppressive function of the T_reg_ population ex vivo.
- Oxidative stress had no effect on the proliferation of the T_reg_ subpopulation or CD11b^+^ myeloid cells in vitro.
- Telomerase function was not required for the ability of T_regs_ to protect against atherosclerosis in vivo; however, telomere length is critical for T_reg_ function.
