# Supplementary material for: Telomerase Mediates Lymphocyte Proliferation but Not the Atherosclerosis-Suppressive Potential of Regulatory T-Cells
Source: Arterioscler Thromb Vasc Biol. 2018 May 29;38(6):1283–96. doi: 10.1161/ATVBAHA.117.309940 (PMC5965929; doi:10.1161/ATVBAHA.117.309940)
Supplement: Supplementary file 1 [file atv-38-1283-s001.pdf]

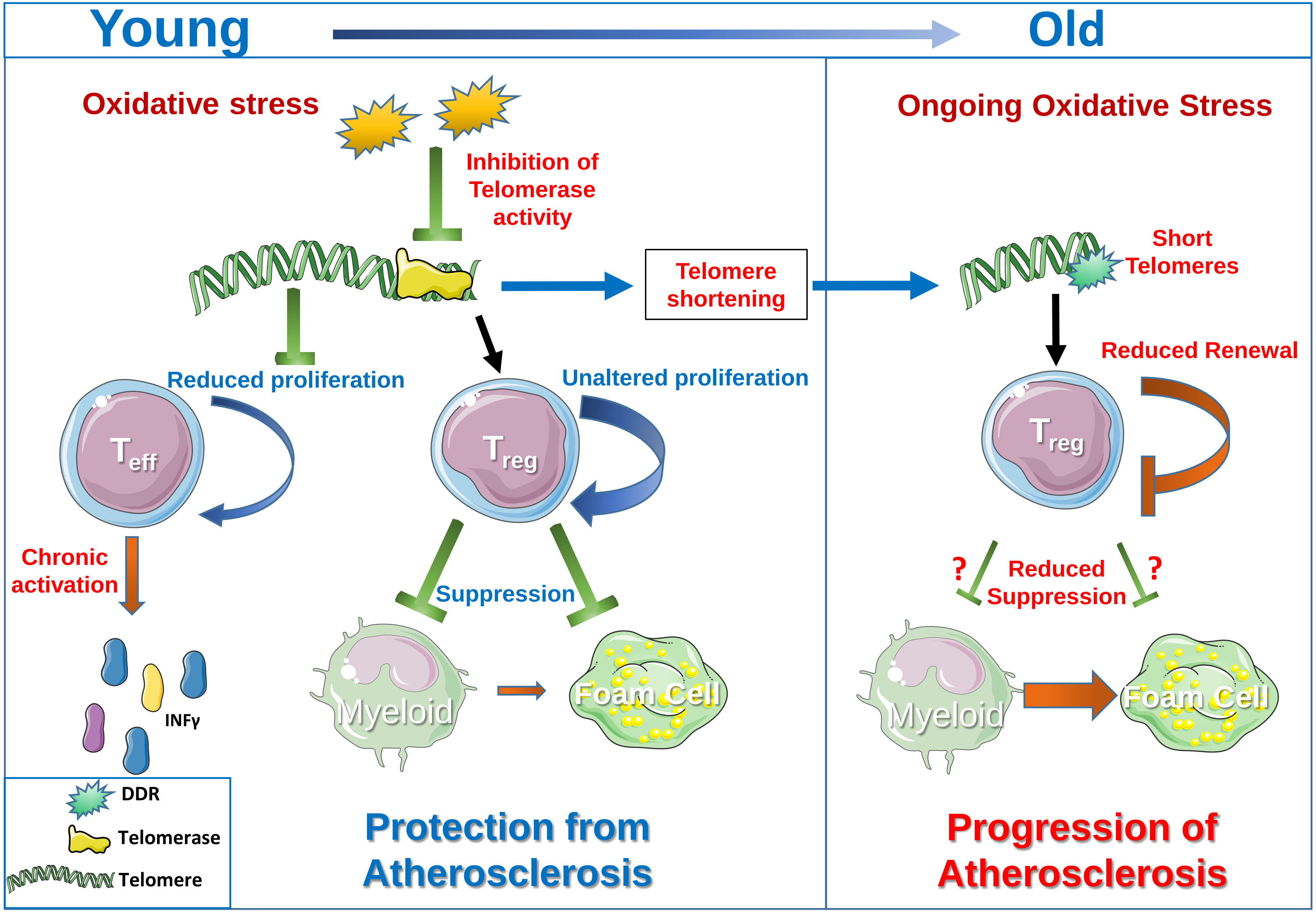
Young
Old
Oxidative stress
Inhibition of Telomerase activity
Telomere shortening
Reduced proliferation
Unaltered proliferation
Teff
Treg
Chronic activation
INFγ
Suppression
Myeloid
Foam Cell
DDR
Telomerase
Telomere
Protection from Atherosclerosis
Ongoing Oxidative Stress
Short Telomeres
Reduced Renewal
Treg
?
Reduced Suppression
?
Myeloid
Foam Cell
Progression of Atherosclerosis
