# Supplementary material for: Telomerase Mediates Lymphocyte Proliferation but Not the Atherosclerosis-Suppressive Potential of Regulatory T-Cells
Source: Arterioscler Thromb Vasc Biol. 2018 May 29;38(6):1283–96. doi: 10.1161/ATVBAHA.117.309940 (PMC5965929; doi:10.1161/ATVBAHA.117.309940)
Supplement: Supplementary file 2 [file atv-38-1283-s002.pdf]

Major Resources Tables

Animals

Species/Strain	Vendor or Source	Background Strain	Sex
Wild type	Charles river	C57BL/6J	Male and Female
B6.129S-Tert, tm1Yjc/J ($TERT^{-/-}$)	Jackson Laboratory	C57BL/6J	Male and Female
Cg-Terc tm1Rdp/J ($TERC^{-/-}$)	Jackson Laboratory	C57BL/6J	Male and Female
mTert-GFP	David Breault Harvard.	C57BL/6J. 129S	Male and Female
$Rag2^{-/-}$ $ApoE^{-/-}$ double knock out	Charlies River	C57BL/6J	Male and Female
$CD28^{-/-}$	Charlies River	C57BL/6J	Male and Female

Antibodies

Target antigen	Vendor or Source	Catalog #	Working concentration	Lot # (preferred but not required)
anti-CD4 (L3T4). For Isolation	Miltenyi Biotec	130-049-201	As per manufacture instructions	
MACSibead mouse T-cell expansion beads	Miltenyi Biotec	130-093-627	As per manufacture instructions	
Anti CD-3. For T cell activation	BD Biosciences	BD 553238	0.5ug/ml	
Anti CD-28. For T cell activation	BD Biosciences	BD 553295	0.5ug/ml	
CD45R (PerCP)	BD Biosciences	553093	0.1 µg/ml	
CD11b (APC)	BD Biosciences	561690	0.1 µg/ml	
CD4 (PE-Cy)	BD Biosciences	552775	0.1 µg/ml	
CD8 (Pacific Blue)	BD Biosciences	558106	0.1 µg/ml	
T_{reg} Detection Kit	Miltenyi Biotec	130-094-164	As per manufacture instructions	
$CD4^{+}CD25^{+}$ T_{reg} isolation kit	Miltenyi Biotec	130-091-041	As per manufacture instructions	
CD4	Biolegend	100531	1µg/ml	
CD25	Biolegend	102012	1µg/ml	
CD3	Biolegend	100321	2µg/ml	
CD62L	Biolegend	104424	2.5µg/ml	
CD44	Biolegend	103012	0.5µg/ml	
Foxp3	Life Technologies	12-5773-82	2µg/ml	
