# Supplementary material for: Telomerase Mediates Lymphocyte Proliferation but Not the Atherosclerosis-Suppressive Potential of Regulatory T-Cells
Source: Arterioscler Thromb Vasc Biol. 2018 May 29;38(6):1283–96. doi: 10.1161/ATVBAHA.117.309940 (PMC5965929; doi:10.1161/ATVBAHA.117.309940)
Supplement: Supplementary file 3 [file atv-38-1283-s003.pdf]

SUPPLEMENTAL MATERIAL

Supplementary Figure I

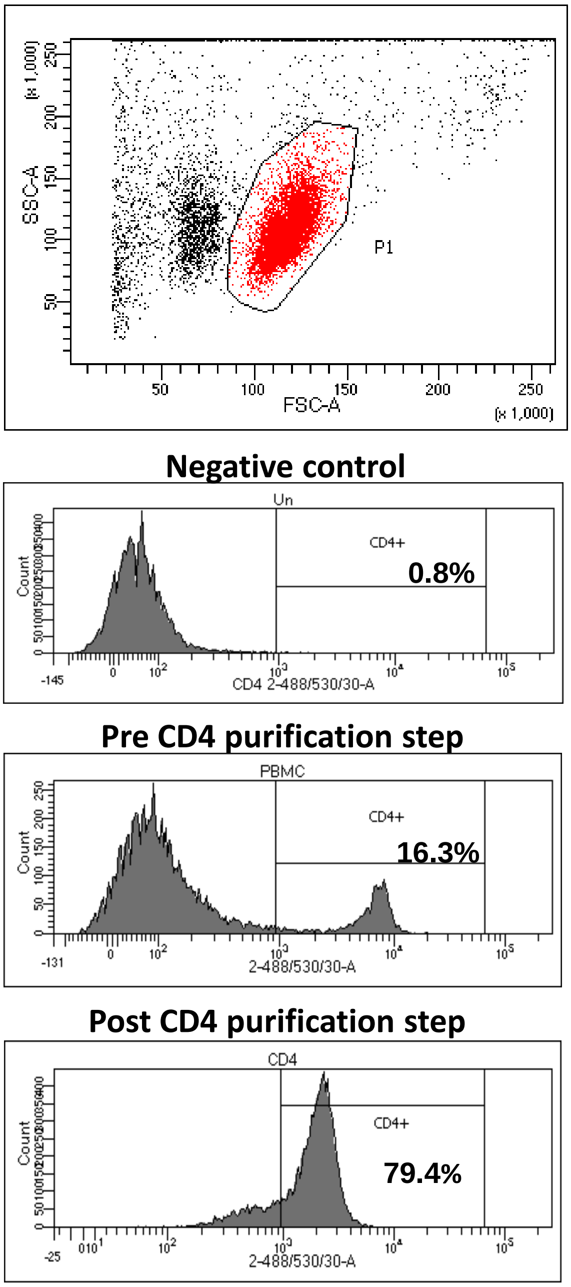

Supplementary Figure I. Example of assessment of CD4 cell purity after isolation CD4 magnetic sorting.

Supplementary Figure II

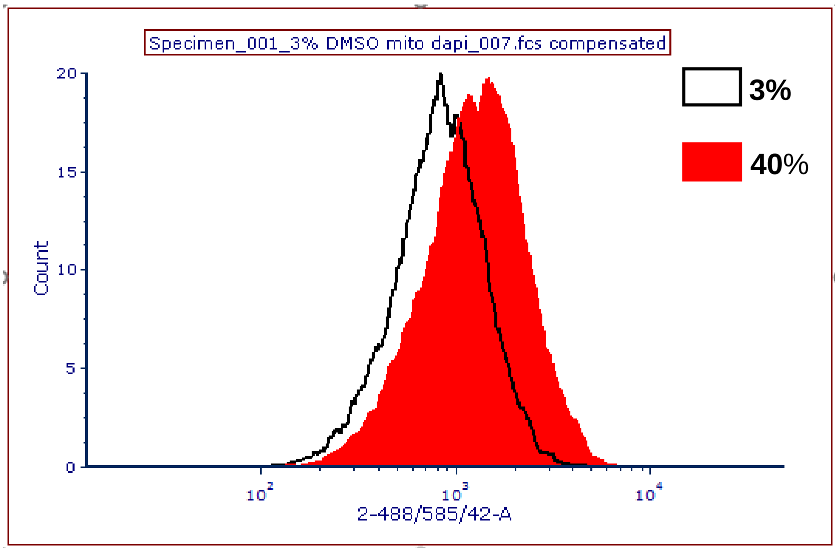

Supplementary Figure II. Hyperoxic culture leads to increased mitochondrial superoxide production. Total splenocytes were cultured at either 3% or 40% oxygen saturation for 6 days. Mitosox fluorescence was increased under hyperoxia compared to cells cultured under 3% oxygen as indicted by a shift in the mean and geomean values when analysed by flow cytometry.

Supplementary Figure III

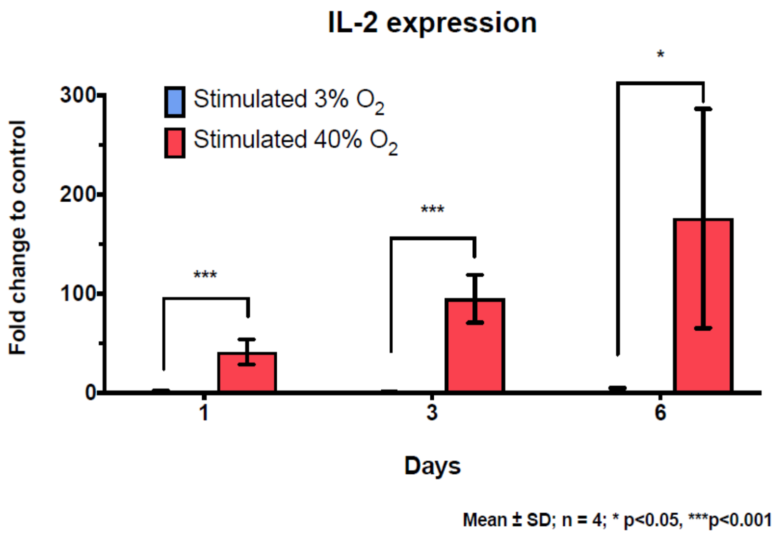

Supplementary Figure III. Oxidative stress leads to increased IL2 expression. CD4+ splenocytes were cultured at $2x10^5$ cells/well under 3% or at 40% oxygen conditions for up to 6 days. IL2 transcript expression was significantly increased at 1, 3 and 6 days under hyperoxia as quantified by qRT-PCR. All error bars represent the standard deviation N>4 for each experimental condition. *P<0.05, **P<0.01, ***P<0.001 and ****P<0.0001 using A 2-WAY ANOVA.

Supplementary Figure IV

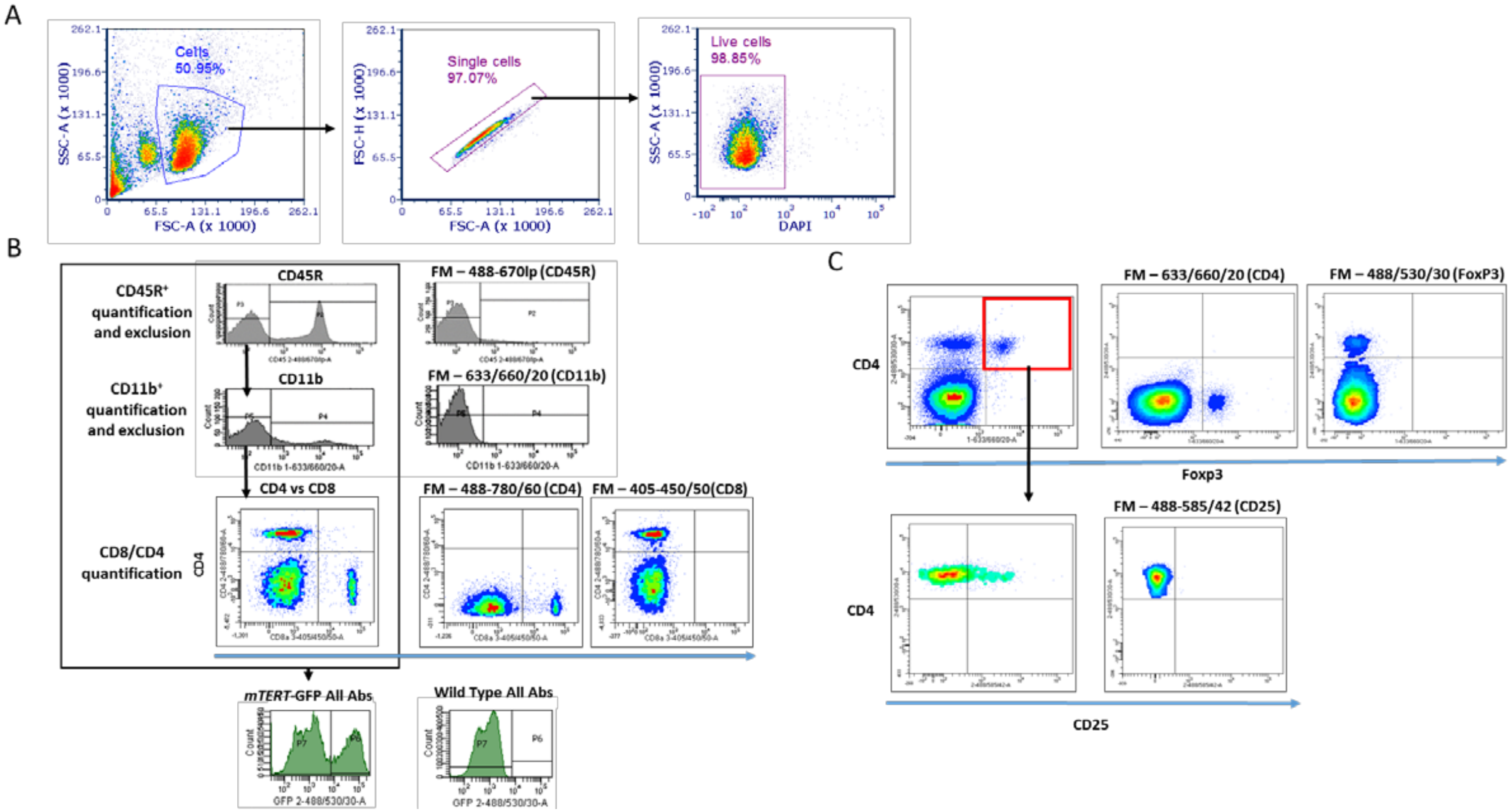

Supplementary Figure IV. Gating strategies. A) Cells were first discriminated from debris based on SSC vs FSC. FSC-H vs FSC-A was used to establish a singlet gate. Cells within the singlet gate were assessed for viability (DAPI exclusion). **B)** Gating strategy for quantification of $CD4^+$/$CD8^+$ T-cells, B cells ($CD45R^+$), myeloid cells ($CD11b^+$) and *mTert*-GFP. Quantification of subpopulations performed as shown. Gating set to florescence minus one controls (FMO). In some experiments *mTert*-GFP was quantified within each subpopulation following the initial gating. Wild type splenocytes stained with all antibodies were used to establish positive gate for GFP expression. **C)** Quantification of T_{reg} populations isolated directly from spleens (As in Figure 4 A-C and Figure 6A). $CD4^+CD8^+$ cells where identified using florescence minus one (FMO) controls to set gating. For these plots the heading indicates the channel in which the absent fluorophore would have been detected and the name of the absent antibody. $CD25^{+/-}$, $CD25^+$ and $CD25^-$ where quantified as a percentage of the $CD4^+CD8^+$ population.

Supplementary Figure V.

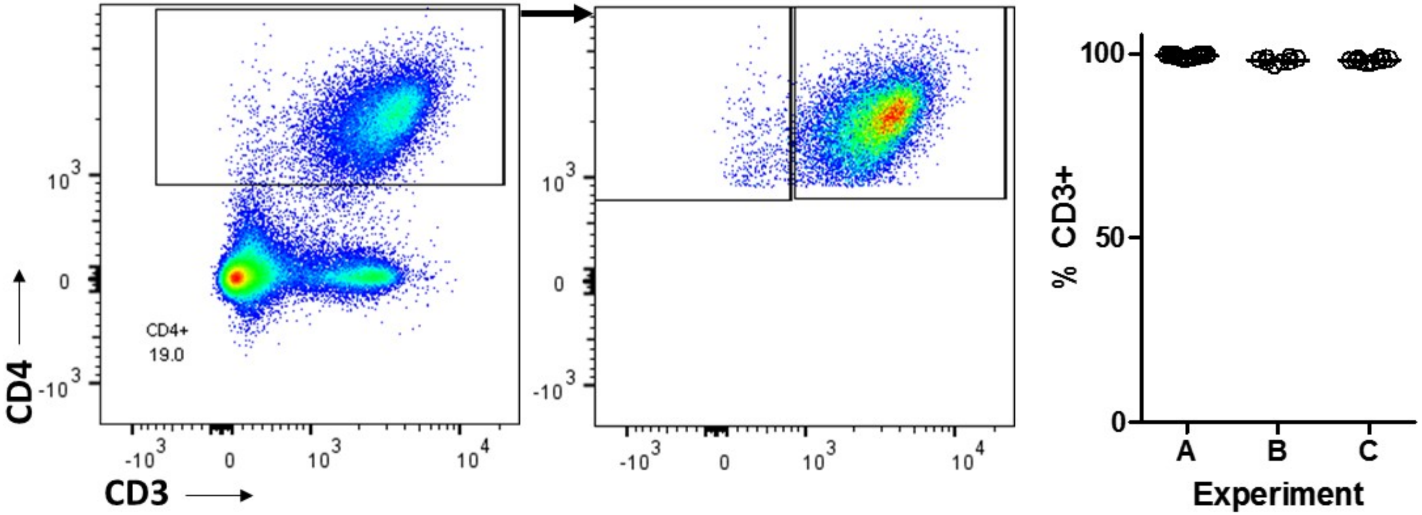

Supplementary Figure V. Quantification of the percentage of $CD3^+$ T-Cells in the $CD4^+$ population in mouse spleen.

Supplementary Figure VI.

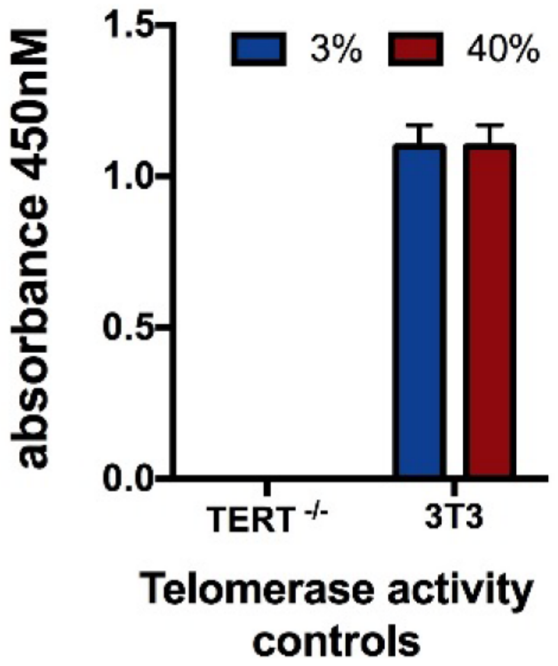

Supplementary Figure VI. TRAP assay controls. Positive control: telomerase expressing immortal fibroblast cell line 3T3. For negative control: Splenocytes isolated from the Tert$^{-/-}$ knockout mouse line.

Supplementary Figure VII

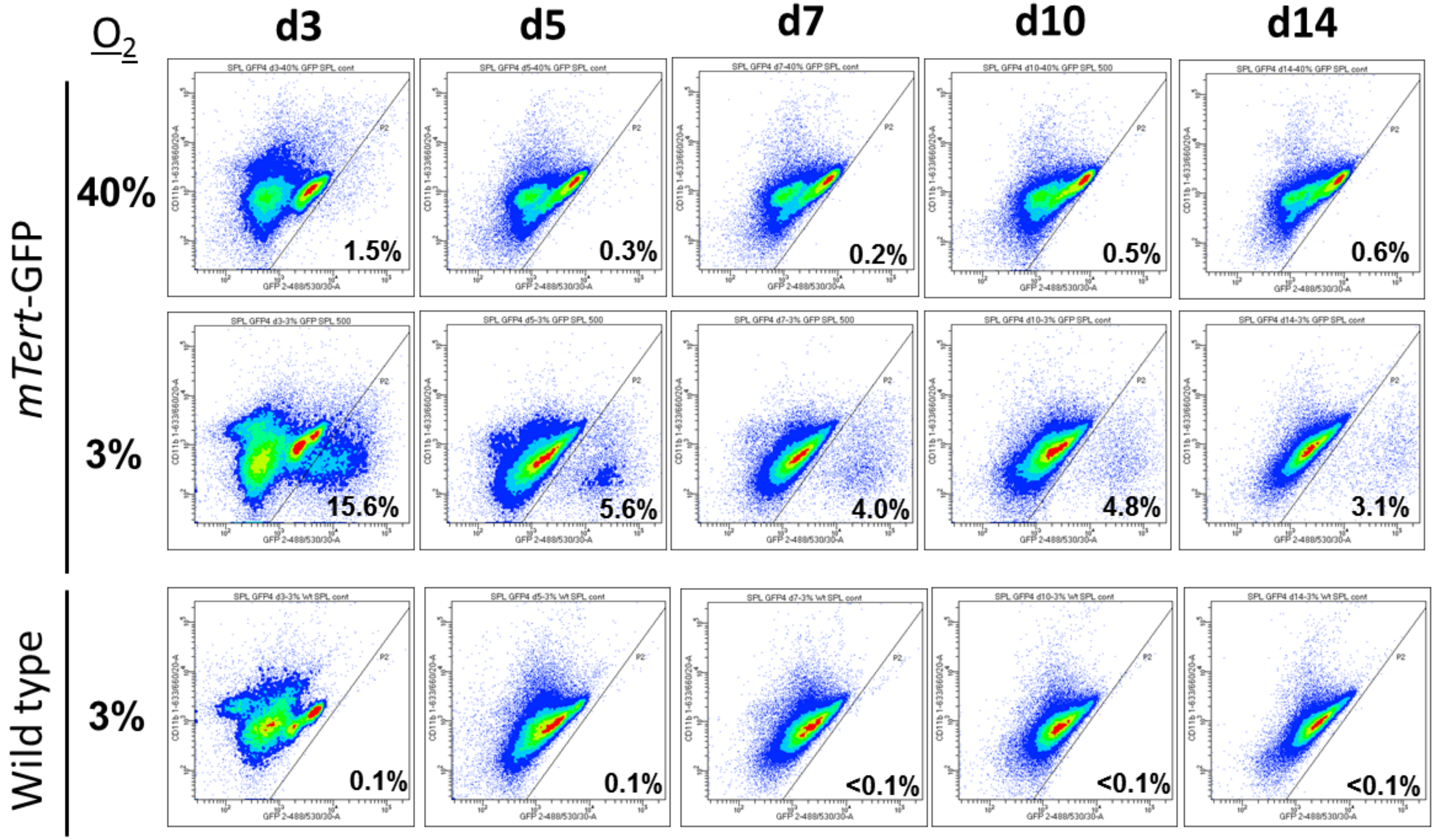

Supplementary Figure VII. Oxidative stress suppresses telomerase at the level of mTert transcription. Splenocytes were isolated from *mTert*-GFP reporter mice and cultured for 14 days at 3% or 40% and T-cell activation maintained with antibody coated plates. *mTert*-GFP expression was quantified at a single cell level on days 3, 4, 7, 10 and 14 days by flow cytometry. Representative flow dot plots for the percentage of total *mTert*-GFP expressing cells at each time-point and condition as a percentage of total cells**.** Gating was established using wild type mice as controls with <0.1% positive events in the mTert-GFP$^+$ gate.

Supplementary Figure VIII.

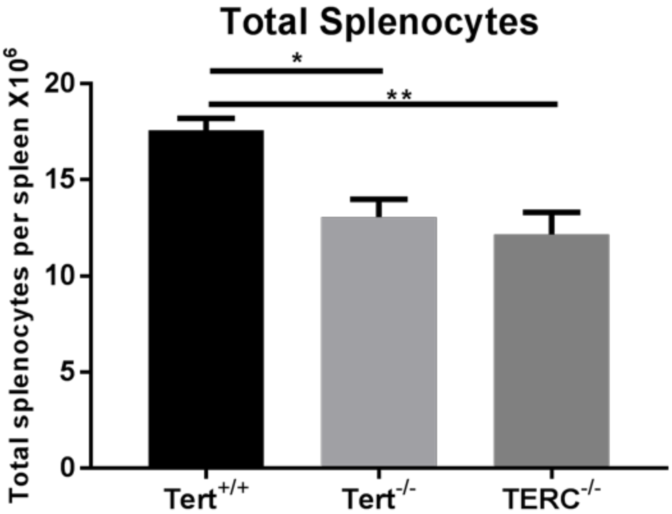

Supplementary Figure VIII. Absolute numbers of Splenocytes obtained during the digestion of each individual spleen. Following digestion viable cells where counted on a hemocytometer using Trypan blue exclusion to distinguish live cells. N>3 for each experimental condition. *P<0.05, **P<0.01 using a 1-WAY ANOVA.

Supplementary Figure IX

Isolated T_{eff} cells ($CD4^{+}CD25^{-}$)

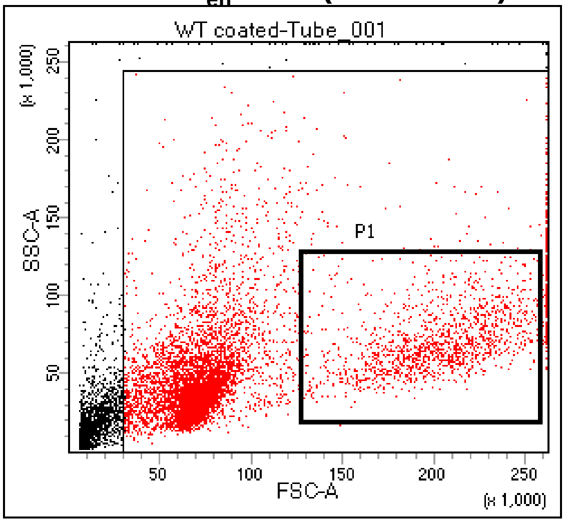

No T-cell activation "positive" control

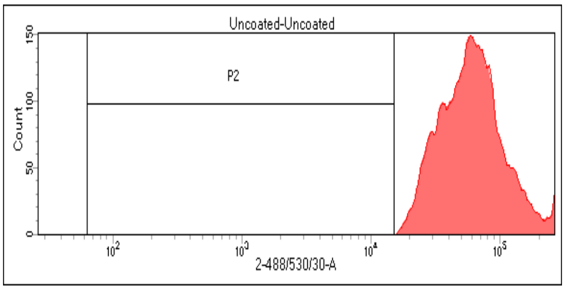

CFSE hi/lo discrimination

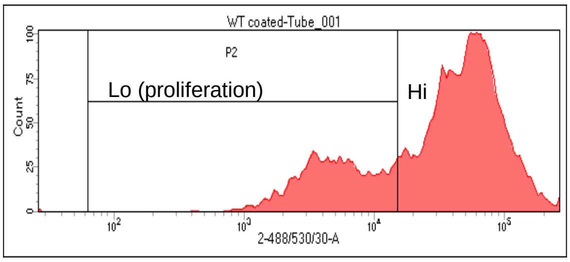

Supplementary Figure IX. Representative flow plots for CFSE proliferation assay.

Supplementary Figure X

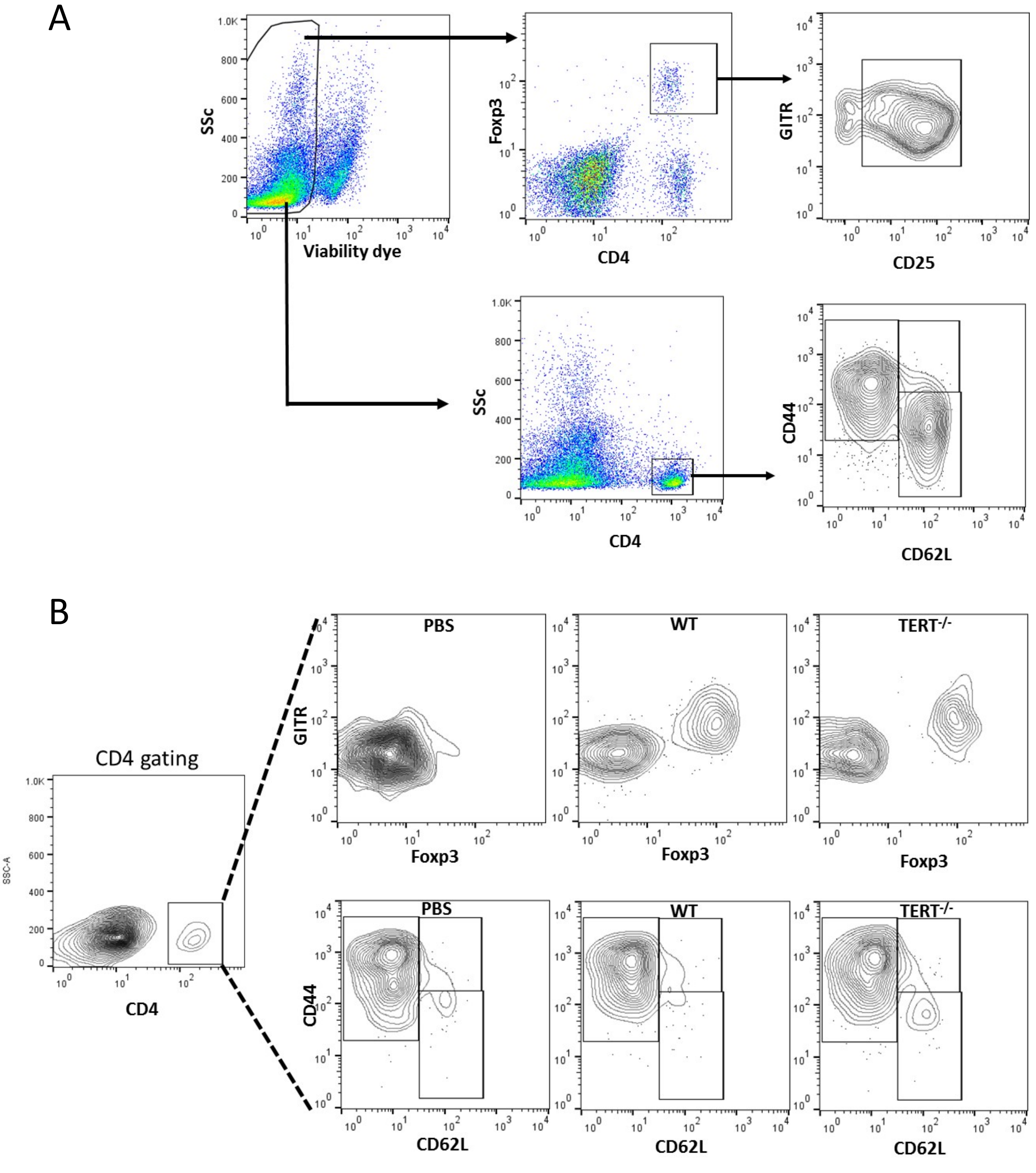

Supplementary Figure X. Gating strategies A) Identification and quantification of T_{reg} (CD4+ GITR+ Foxp3^{+}) or T_{eff} (CD4^{+} CD62^{-} CD44^{hi}) in Rag2$^{-/-}$ mice. Rag2$^{-/-}$ mice were transplanted with CD28$^{-/-}$ splenocytes and either wild type T_{reg},TERT$^{-/-}$ T_{reg} cells or PBS as control (no transplanted T_{regs}). Cells isolated from either spleen or lymph nodes where gated for viability (DAPI). **A)** Viable cells were either i) analysed for Foxp3 vs CD4 antibody labelling or ii) Gated based on CD4 antibody staining followed by subsequent analysis for CD44 and CD62L. **B)** Representative example of flow cytometry plots of T_{reg} (CD4^{+} GITR^{+} Foxp3^{+}) or T_{eff} (CD4^{+} CD62^{-} CD44^{hi}) found in rag2$^{-/-}$ mice transplanted with CD28$^{-/-}$ splenocytes and either wild type T_{reg}, TERT$^{-/-}$ T_{reg} cells or PBS control (no transplanted T_{regs}).

Supplementary Table I Details of mouse strain and genetics used in each study.

Figure	Subfigure	Strain	Genetics
**1**	A	C57BL/6J	Wild type
	B	C57BL/6J	Wild type
	C	C57BL/6J	Wild type
	D	C57BL/6J	Wild type
	E	C57BL/6J	Wild type
	F	C57BL/6J	Wild type
**2**	A	B6.129S-Tert, tm1Yjc/J	Wild type littermates **F1**
	B	B6.129S-Tert, tm1Yjc/J	$TERT^{-/-}$ **F1**
	C	B6.129S-Tert, tm1Yjc/J	Wild type littermates **F1**
	D	B6.129S-Tert, tm1Yjc/J	$TERT^{-/-}$ **F1**
	E	B6.129S-Tert, tm1Yjc/J	Wild type litter mates **F1**
	F	B6.129S-Tert, tm1Yjc/J	$TERT^{-/-}$ **F1**
	G	C57BL/6J	Wild type
	F	C57BL/6J	Wild type
**3**	A	B6.129S	*mTert*-GFP
	B	B6.129S	*mTert*-GFP
	C	B6.129S	*mTert*-GFP
**4**	A	B6.129S-Tert, tm1Yjc/J	$TERT^{-/-}$ **F1** or Wild type litter mates
	B	B6.129S-Tert, tm1Yjc/J	$TERT^{-/-}$ **F1** or Wild type litter mates
	C	B6.129S-Tert, tm1Yjc/J	$TERT^{-/-}$ **F1** or Wild type litter mates
	D	T_{regs} = B6.129S-Tert, tm1Yjc/J<br><br>T_{eff} = B6.129S-Tert, tm1Yjc/J	T_{regs} = $TERT^{-/-}$ **F1** or Wild type litter mates<br><br>T_{eff} = Wildtype =litter mates
	E	T_{regs} = B6.129S-Tert, tm1Yjc/J<br><br>T_{eff} = B6.129S-Tert, tm1Yjc/J	T_{regs} = $TERT^{-/-}$ **F1** or Wild type litter mates<br><br>T_{eff} = Wildtype litter mates
	F	C57BL/6J	Wild type

**5**	A -G	Recipient mice = C57BL/6<br><br>CD28$^{-/-}$ Donor mice = C57BL/6J<br><br>Tregs = B6.129S	Recipient mice = Rag2$^{-/-}$ /ApoE$^{-/-}$<br><br>CD28$^{-/-}$ Donor mice = CD28 knock out<br><br>Tregs = TERT$^{-/-}$ **F1** or Wild type litter mates
**6**	A	B6.Cg-Terc, m1Rdp/J (Blasco et al.,131 1997).	TERC$^{-/-}$ **F1**or Wild type litter mates
	B	B6.Cg-Terc, m1Rdp/J (Blasco et al.,131 1997).	TERC$^{-/-}$ **F1** or Wild type litter mates
	C	B6.Cg-Terc, m1Rdp/J (Blasco et al.,131 1997).	TERC$^{-/-}$ **F1** or Wild type litter mates
